# Vaccines in 2025: What Is New in Vaccine Advocacy?

**DOI:** 10.3390/vaccines14010060

**Published:** 2026-01-04

**Authors:** Lavinia Bianco, Christian Napoli

**Affiliations:** 1 Department of Medical Surgical Sciences and Translational Medicine, Sapienza University of Rome, 00189 Rome, Italy; lavinia.bianco@uniroma1.it; 2 National Institute for Health, Migration and Poverty (NIHMP), 00153 Rome, Italy

The scientific literature is quite rich when it comes to vaccines and vaccination: just by looking at PubMed, using as “Vaccin* [Title/Abstract]” a search key, it is possible to obtain more than 480,000 results starting from 1799, and more than 25,288 results were published in the last year. The availability of this incredible amount of literature is due to the incredible importance of the subject, as widespread use of vaccines has led to the global eradication of smallpox and near-eradication of polio, measles, rubella and neonatal tetanus [1,2]. Moreover, research in this field is very proactive: as of 21 March 2025, the global landscape of vaccine R&D included 919 candidates identified since 2015, with a heavy focus on COVID-19, influenza, HIV, HPV, pneumococcal and RSV vaccine candidates [3].

In short, it is possible to state that vaccination has revolutionized the way in which we prevent and control infectious diseases [2], and the continuous search for both proof of their usefulness and new technologies is the main reason that this journal was launched.

As expected, vaccines received significant consideration during the COVID-19 pandemic, as the whole world paid attention to equitable access to vaccines, vaccine supply and logistics (such as information systems for tracking COVID-19 vaccination and monitoring vaccine safety), educating and empowering health-care professionals at all levels and addressing confidence in and demand for vaccines [1].

In fact, a robust immunization policy, combined with strong advocacy for vaccination, has the potential to increase the resilience of immunization programs [4]; it should always be remembered that both vaccine hesitancy and vaccine acceptance depend, among other factors, on education level and on the information provided about vaccines [5,6]. Notably, a recently published review identified and summarized the current tools available to address vaccine hesitancy and increase uptake, such as the implementation of culturally and linguistically responsive educational campaigns, using trusted messengers through community engagement, and strong provider recommendations [7]. Moreover, even considering the heightened attention given to vaccines during the COVID-19 pandemic, there was no gain for the R&D groups, as no significant differences in the number of vaccine candidates were found across the different periods of the COVID-19 pandemic [3].

In other words, a simple understanding of the benefits of vaccinations for both single individuals and entire communities [2], as well as their indisputable role in reducing morbidity and death from a growing number of infectious and noncommunicable diseases [1], is insufficient if we cannot correctly and efficiently share that knowledge. Knowing, as experts in the field, that not getting vaccinated can lead to the reappearance of previously eradicated illnesses [2] and that better collaborations from academic and nonprofit sectors could aid R&D for infectious diseases vaccines [3] might not be enough to ensure the protection of our very own communities if said communities do not work with us, hence the growing attention given to community engagement.

On this topic, the literature suggests that emphasizing the concept of herd immunity in vaccination advocacy might improve vaccination acceptance [8] and that implementing measures such as school-based education programs, achieving visible leadership influence and using various media channels could be useful for accurate vaccine information dissemination [9]. Moreover, communication must be tailored: for instance, pediatricians and other clinicians who care for children must be ready to answer parents’ questions and doubts regarding vaccine licensure, vaccine safety and vaccine safety monitoring, individually identifying and addressing each parental vaccine concern [7,10], whereas general practitioners must also answer questions regarding vaccine development [11].

In conclusion, guaranteeing the correct vaccination and immunization of a community is a group effort, and evidence gathered in the last 5 years proves, once again, that effective communication strategies are necessary for addressing vaccine hesitancy [7,10,11]. To keep up with the new technologies and to assure strong advocacy for vaccination towards the general population, it is crucial to monitor this ever-growing body of evidence.
